# Predicting the fungal CUG codon translation with Bagheera

**DOI:** 10.1186/1471-2164-15-411

**Published:** 2014-05-29

**Authors:** Stefanie Mühlhausen, Martin Kollmar

**Affiliations:** Group Systems Biology of Motor Proteins, Department of NMR-based Structural Biology, Max-Planck-Institute for Biophysical Chemistry, Göttingen, Germany

## Abstract

**Background:**

Many eukaryotes have been shown to use alternative schemes to the universal genetic code. While most *Saccharomycetes*, including *Saccharomyces cerevisiae*, use the standard genetic code translating the CUG codon as leucine, some yeasts, including many but not all of the “*Candida*”, translate the same codon as serine. It has been proposed that the change in codon identity was accomplished by an almost complete loss of the original CUG codons, making the CUG positions within the extant species highly discriminative for the one or other translation scheme.

**Results:**

In order to improve the prediction of genes in yeast species by providing the correct CUG decoding scheme we implemented a web server, called Bagheera, that allows determining the most probable CUG codon translation for a given transcriptome or genome assembly based on extensive reference data. As reference data we use 2071 manually assembled and annotated sequences from 38 cytoskeletal and motor proteins belonging to 79 yeast species. The web service includes a pipeline, which starts with predicting and aligning homologous genes to the reference data. CUG codon positions within the predicted genes are analysed with respect to amino acid similarity and CUG codon conservation in related species. In addition, the tRNA_CAG_ gene is predicted in genomic data and compared to known leu-tRNA_CAG_ and ser-tRNA_CAG_ genes. Bagheera can also be used to evaluate any mRNA and protein sequence data with the codon usage of the respective species. The usage of the system has been demonstrated by analysing six genomes not included in the reference data.

**Conclusions:**

Gene prediction and consecutive comparison with reference data from other *Saccharomycetes* are sufficient to predict the most probable decoding scheme for CUG codons. This approach has been implemented into Bagheera (http://www.motorprotein.de/bagheera).

**Electronic supplementary material:**

The online version of this article (doi:10.1186/1471-2164-15-411) contains supplementary material, which is available to authorized users.

## Background

For a long time it is known that many organisms show alterations to the universal genetic code [1, 2]. These codon reassignments could have happened under strong AT or GC pressure, which might lead to the complete disappearance of the reassigned codon followed by a tRNA with a different amino acid identity taking over the decoding of the respective codon during its reappearance (“codon capture” theory [3]). In a mutually exclusive scenario, the codon is in a transitional state, in which it is decoded ambiguously by two tRNAs (“ambiguous intermediate” theory [4]). An example for the latter scenario is the reassignment of the CUG codon from leucine to serine in *Candida* yeasts [5–7], which cannot be accomplished by a single mutation in the anticodon of a serine tRNA. Indeed, *Candida* species contain a single tRNA with a CAG anticodon (Ser-tRNA_CAG_) [8].

The general time line of the switch in using the leucine CUG codon for serine in fungi has already been investigated. Shortly, the unusual Ser-tRNA_CAG_ appeared about 270 million years (Ma) ago. However, the genera *Candida* (CUG codes for serine) and *Saccharomyces* (CUG codes for leucine) separated from each other about 180 Ma ago implying that the codon ambiguity remained for about 100 Ma in the ancestors of the yeasts [8, 9]. The ancestor of the *Saccharomyces* lost the mutant Ser-tRNA_CAG_ and retained the wild-type Leu-tRNA_CAG_, while the ancestor of the *Candida* lost the Leu-tRNA_CAG_ and maintained the mutant Ser-tRNA_CAG_ changing the identity of the CUG codon from leucine to serine. A whole genome comparison showed that only a minor fraction of the CUG codons present in *Candida albicans* have equivalent CUGs in *Saccharomyces cerevisiae* implying that almost all original CUG codons disappeared in *C.albicans*[9].

However, the decoding cannot be derived unambiguously from the species names (e.g. “*Candida*” species exist all over the yeast tree [10–13]). In several taxonomically broad protein family analyses [14–16] we have observed that CUG positions are conserved within many of these sequences, that many mapped to structurally conserved residues and can thus often unambiguously assigned to either leucine (large hydrophobic residue, at alignment positions highly enriched in hydrophobic residues) or serine (small polar residue). These observations also suggest that the data can be used as reference for the assignment of the CUG codon translation to further sequenced yeast species in the future.

Here, we provide a tool with which it can fast and easily be determined whether a yeast species uses the Standard Codon Usage or the Alternative Yeast Codon Usage (AYCU). The tool is suitable for both data from whole genome projects and transcriptome analyses. The tool is also thought to provide a reference page for species using the AYCU. In addition, the tool allows easy examination of the correct decoding of existing annotated genes by translating mRNA using the Standard or Alternative Yeast Codon Usage and by verifying the translation of a given protein sequence via gene reconstruction in the respective species. The tool closes an important gap in yeast research because the NCBI taxonomy does not reflect the latest phylogeny, and the assignment of the genetic code at the NCBI webpages is wrong for many species. E.g. *Lodderomyces elongisporus*, *Hyphopichia burtonii*, *Candida tenuis*, and others are denoted as using the Standard Code instead of the AYCU, which is known for e.g. *Lodderomyces*[17] and *C.tenuis*[7] for many years.

## Implementation

### Technologies

The system has been developed to run on Linux systems. The web application is implemented in the Ruby programming language (version 1.9.3; [18]) using the Ruby on Rails framework (version 3.2.12; [19]), which has the advantage of rapid and agile development while keeping the code well organized. The site makes extensive use of Ajax (Asynchronous JavaScript and XML) in order to present the user with a feature rich interface while minimizing the amount of transferred data. The alignments and phylogenetic trees are visualized with the Lucullus software, which is a plugin to PyBioMaps [20]. Performance is enhanced by parallelization of the prediction process. All technologies used are freely available and open source.

### Workflow

**Figure 1:**
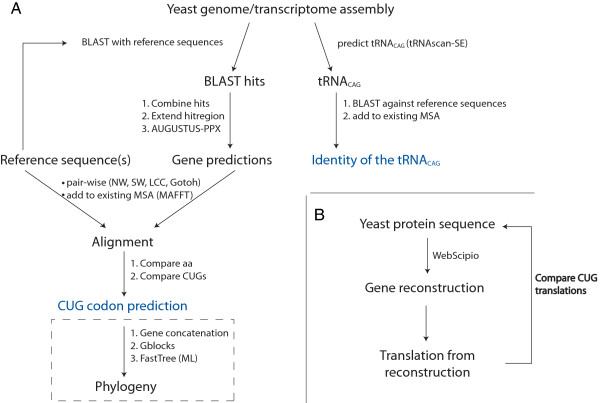
**Workflow of the Bagheera web application. A)** Upon uploading of the yeast genome or transcriptome assembly data homologous proteins to the reference sequences are identified using TBLASTN and subsequently predicted by AUGUSTUS-PPX. The reference sequences used for the gene prediction are selected according to the species selected as model organism for AUGUSTUS. The predicted proteins are aligned to the reference alignments (NW = Needleman-Wunsch, SW = Swith-Waterman, LCS = Longest Common Subsequence) and the codon usage predicted based on the analysis of sequence similarity and CUG codon conservation at CUG codon positions. Optionally, a phylogenetic tree can be calculated based on a randomly selected and concatenated subset of the predicted proteins. **B)** A gene reconstruction of the uploaded protein sequence is performed to obtain cDNA sequence. The species encoding the uploaded protein has to be specified. The cDNA sequence is then translated according to the translation scheme of the respective species.

The implementation of the CUG prediction workflow is shown in Figure 1A. The uploaded yeast genome or transcriptome assembly data is searched with representatives of 2071 proteins from 38 different protein families and classes using TBLASTN [21, 22]. The protein reference dataset is updated from CyMoBase [23, 24] on a monthly basis. The genomic regions of the BLAST hits are extended by 500 nucleotides in both directions to obtain better and more complete *ab initio* gene predictions. Subsequently, overlapping BLAST hits are combined because they presumably belong to the same gene. Genes within these extended genomic regions are predicted by AUGUSTUS-PPX [25, 26] with the options --genemodel=exactlyone to predict exactly one gene, --proteinprofile to integrate pre-calculated profiles for each protein family of the reference data, and --species_model to use species-specific parameters. Subsequently, the AUGUSTUS predictions are aligned to the reference multiple sequence alignments with MAFFT [27], or to the reference sequence used for the TBLASTN search with C++ implementations of the Needleman-Wunsch [28], Gotoh [29], Smith-Waterman [30] or Longest Common Subsequence [31] algorithms, which are part of the SeqAn algorithm library [32]. CUG positions within the predicted genes are then compared to the reference data with respect to amino acid conservation and, if existing, to the translations of CUG codons at the same position in the reference data. Optionally, the user can compute the phylogenetic grouping of the yeast query data to the reference data. For this purpose, five or ten of the predicted sequences are chosen randomly, the corresponding alignments of the gene predictions and reference data are concatenated, poorly aligned positions are removed with Gblocks v.0.91b [33], and the phylogenetic tree is computed with FastTree [34]. In addition to this sequence-based prediction of the translation scheme, the identity of the tRNA_CAG_ is predicted (Figure 1A). For this purpose, tRNAs and their secondary structures are predicted with tRNAscan-SE under a general (−G) or a eukaryote-specific tRNA model [35]. Pseudogene checking is disabled with option –D to speed up the search process. The predicted tRNA_CAG_ is subsequently compared to reference data consisting of 51 leu-tRNA_CAG_ genes, 22 ser-tRNA_CAG_ genes, and 34 tRNA genes with other anticodons. For visual inspection, the predicted tRNA_CAG_ is aligned to the reference tRNA data with MAFFT.

The workflow for the verification of the CUG translation in a given protein sequence is shown in Figure 1B. Shortly, a gene reconstruction of the query protein in the selected species is performed with WebScipio [36]. Optionally, the gene reconstruction can be performed with less stringent parameters, which include relaxed values for the parameters --minimal identity, −-maximal mismatches and --minimal score to allow prediction of less similar genes. This option is very useful if the respective query gene has not been derived from one of the reference species but a closely related one. The coding DNA obtained from the gene reconstruction is re-translated into protein sequence using the translation scheme of the selected species, which is already implemented in WebScipio.

For the translation of a provided mRNA into protein, the mRNA is first split into codons, which are then translated using the specified translation scheme. Extra nucleotides are ignored.

### Identification and annotation of the reference data

Fungal actin and actin-related proteins, dynactin proteins, myosins, kinesins, dynein heavy chains, tubulins, actin-capping proteins CapZ, coronins and WASP homologs have been extracted from previously published datasets [14–16, 37]. The sequences were updated based on newer genome assemblies if necessary. The reference data for the other proteins and the species not included in the published datasets have essentially been obtained as described in [14]. Shortly, the corresponding genes have been identified in TBLASTN searches starting with the respective protein sequence of homologs of *Saccharomyces cerevisiae*. The respective genomic regions were submitted to AUGUSTUS [25] to obtain gene predictions. However, feature sets are only available for a few species of the *Saccharomycetes* clade. Therefore, all hits were subsequently manually analysed at the genomic DNA level. When necessary, gene predictions were corrected by comparison with the homologs already included in the multiple sequence alignments.

Reference tRNA_CAG_ genes were predicted with tRNAscan-SE in 45 yeast species. Intron regions were removed and tRNAs aligned manually.

All sequence related data (protein names, corresponding species, sequences, and gene structure reconstructions) and references to genome sequencing centres are available at CyMoBase (http://www.cymobase.org, [23]). A list of the reference species and their abbreviations as used in the alignments and trees, as well as anamorph and alternative names can be accessed through the web server and as Additional file 1. Additional file 1 also includes references to published genomes, and detailed information and acknowledgments of the respective sequencing centres. All gene structures for the reference dataset have been reconstructed with Scipio/WebScipio [36, 38].

### Results and discussion

The first step in gene annotation is gene prediction, which can be done in a genome-wide scan or for single genes. Many gene prediction programs allow using different codon translation tables, but this option is not available in most of the gene prediction web interfaces. Even then, it would require the user to know a priori, whether the target organism belongs to the species not using the standard codon table. Especially the yeasts are confusing, as the *“Candida”* species are well known to use the AYCU in contrast to *Saccharomyces cerevisiae*. But this has only been shown for a few *Candida* species, including some of the most pathogenic, and many yeast species are called *Candida* although there is no monophyletic “*Candida* clade”. Codon decoding schemes cannot unambiguously be derived from single-gene studies because the respective gene might not contain the codon in question at all, or the respective amino acids are not at meaningful positions. Meaningful positions would be those that are strongly conserved in evolution and therefore in the core of the proteins or at conserved binding interfaces at the surface. In the course of our continuous efforts in identifying and annotating cytoskeletal and motor proteins [14–16, 37] we have already assembled and annotated 2071 sequences from 18 protein families in 79 yeast species. Some of the data has already been used to evaluate the CUG encoding in 60 completely sequenced yeasts (Mühlhausen and Kollmar, unpublished data). Here, these data are used as reference dataset in a pipeline for the prediction of the CUG codon translation, which can be accessed by users through a web interface.

### The reference data

Currently, CyMoBase [23, 24], a database for manually assembled and annotated cytoskeletal and motor proteins, contains 26 protein families with annotated proteins in 79 yeast species. All sub-families of these protein families, like for example the α-, β-, and γ-tubulins, already existed in the last common ancestor of the opisthokonts or even the eukaryotes and are therefore treated as independent proteins. Not all protein families in CyMoBase have been analysed at the same depth, e.g. only two dynein light-intermediate chain proteins are available yet. Also, some sub-families like the class-17 myosins or the class-4 kinesins are only present in early diverging yeast species and not in e.g. *Candida albicans* or *Saccharomyces cerevisiae*. These proteins do not provide the necessary statistical basis and taxonomic sampling for a CUG prediction and were not included in the Bagheera reference data. Bagheera’s reference data thus consists of 18 protein families (38 independent proteins) comprising 2071 sequences. These data will increase in the future in the course of our continuous efforts in annotating cytoskeletal and motor proteins. Most of the reference proteins are considerably longer then the average yeast proteins like for example the myosins (1100 to 2400 amino acids) and the dynein heavy chain proteins (about 4000 amino acids), and the reference data therefore comprises significantly more data than the sole numbers of proteins and sequences might implicate (Additional files 1 and 2).

### The web interface

**Figure 2:**
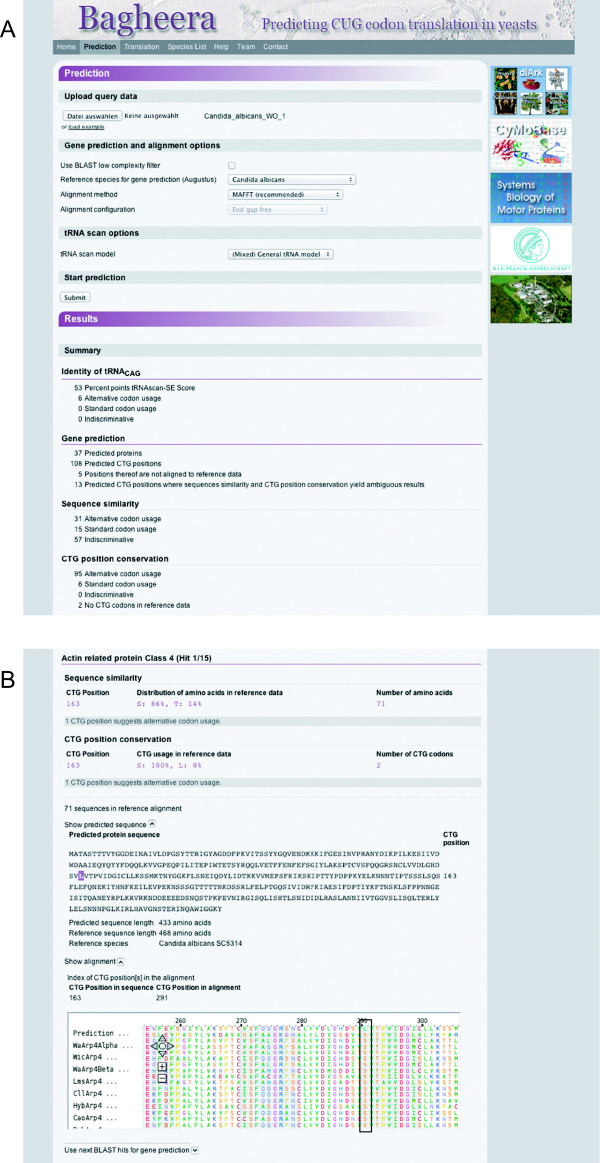
**Screenshot of the web interface.** The web interface is divided into three main parts: data upload and options section, results section, and phylogenetic tree section (not shown). **A)** Example data were uploaded and processed with default parameters. **B)** The results section is split into a summary and a section listing each reference protein and a detailed analysis of each predicted protein down to single CUG codons. For every reference protein, the predicted gene and, if applicable, the respective CUG positions are shown. For every predicted CUG position, which could be mapped onto the reference data, the amino acid composition and CUG codon usage at the respective positions in the reference data are listed. The predicted actin related protein class 4 (Arp4) contains one CUG at position 163. This position corresponds to alignment position 291 in the reference alignment. It is here indicated by a black box. All CUG codons are noted as leucine in the predicted sequence, regardless the suggested codon usage.

Great attention has been paid to a versatile yet easy to use web interface (Figure 2). We think that accessibility and high quality representation is key to a productive usage of the system. Bagheera offers possibilities to analyse large-scale, whole genome and transcriptome assembly data, and to determine the correct CUG translation for any single cDNA or protein sequence.

Regarding the prediction of the most probable CUG translation in large-scale data, Bagheera does the following: i) The user provides genomic data, e.g. a genome assembly, transcriptome assembly or long-read EST data, or data from single to multiple gene analyses. ii) The tool predicts cytoskeletal and motor proteins and aligns the predicted sequences to the respective protein families. iii) The respective positions of the CUG codons of the predicted sequences are compared to the reference data. iv) The tool predicts tRNA_CAG_ and performs a sequence similarity search and sequence alignment with reference leu-tRNA_CAG_ and ser-tRNA_CAG_ genes.

The verification of the translation of a single sequence depends on the provided sequence. The translation of a given cDNA sequence is done as follows: i) The user provides an mRNA sequence and specifies the translation scheme. ii) The tool translates the mRNA into protein. For any given protein sequence the workflow is: i) The user provides a protein sequence and specifies the corresponding species. ii) The tool performs a gene reconstruction for the protein with WebScipio and re-translates the coding regions using the corresponding CUG translation scheme as provided by WebScipio. iii) CUG translations in the user-provided protein are compared to translations in the re-translated.

Care has to be taken when using transcriptome assembly or long-read EST data as input. Transcriptome data do not represent all coding regions of a species, which might lead to wrong assignment of predicted proteins to the reference data. For example, actin and the actin-related proteins are closely related, or the α-, β-, and γ-tubulins, or the members of the other multi-gene protein families. In the case that only actin genes are present in the transcriptome data these would be identified as closest homologs and aligned to all of the actin-related proteins of the reference data. While key residues for folding and ATP-binding are of course conserved between actin and all actin-related proteins, residues in loop regions are less or not at all conserved. Within the same sub-family these regions could also contain valuable information, because loops might be conserved within the entire sub-family. By aligning proteins from different sub-families this information might at best be lost or in the worst case even lead to contradicting results.

In the first step of the prediction pipeline, homologs to the proteins of the reference data need to be identified. Here, one gene is predicted for every protein family present in the reference data using TBLASTN [21] and AUGUSTUS-PPX [25, 26]. We choose BLAST as search algorithm because it is very fast and not as restrictive in terms of sequence homology as for example BLAT [39]. To optimize the search and subsequent gene prediction the user can select one of the AUGUSTUS feature sets, which contain species-optimized parameters and are available for a number of yeast species. The reference proteins used in the BLAST search are taken from the species, which had been selected for the AUGUSTUS prediction. In most cases, the BLAST hits do not cover the entire genes but miss the N- and C-termini, and low complexity regions. In the latter case and in the case that genes are split into several exons, the search results in several BLAST hits belonging to the same gene. These partial hits are combined and extended in the 5' and 3' direction because AUGUSTUS gene predictions are significantly better when intergenic regions are included in the genomic regions.

### Prediction of the CUG codon translation

**Figure 3:**
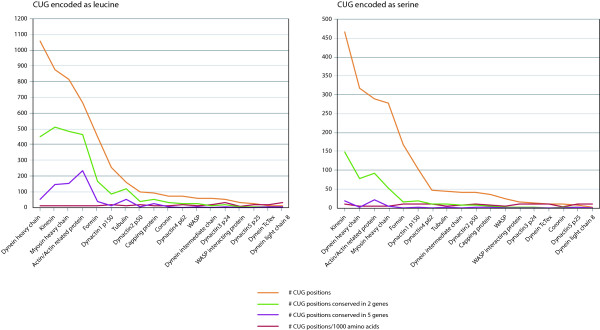
**Number of CUG codons in the reference data.** The total number of CUG positions for every set of reference proteins is shown together with the numbers of CUG positions conserved in at least two and five genes. To account for different protein lengths (e.g. 200 amino acids in dynactin3 p24 proteins compared to up to 4,000 amino acids in dynein heavy chain proteins), the total number of CUG positions per 1,000 amino acids is also plotted showing that CUG codons are not particularly enriched in certain protein families. Values for all species using standard codon usage (left side) are contrasted with those for all species using alternative yeast codon usage (right side). Detailed numbers are available in Additional file 3.

In the second step, the most probable codon usage in the predicted sequences is determined. To this end, the predicted sequences are aligned to the multiple sequence alignments of the respective protein families or, optionally, only to the reference sequence, which has been used in the BLAST search. We choose MAFFT [27] as default alignment method, because it allows adding a new sequence to an already existing multiple sequence alignment. Based on these alignments two features are analysed: CUG position and amino acid conservation. The reference data contains 8244 known CUG codons (Figure 3, Additional file 3), and it is determined whether CUG codons in the query data match CUG codons in the reference data. In addition, the amino acid compositions at alignment positions, where the query data contain CUGs, are determined. The usage of reference genes and their encoding of CUG codons are restricted to completely assembled genes, while the reference amino acid composition is also calculated on basis of incompletely assembled genes. Based on these data, the most probable codon usage for every CUG codon is predicted. Here, the encoding of CUG codons in the reference data as leucine and the presence of hydrophobic amino acids (leucine, isoleucine, valine, methionine, phenylalanine) at the respective alignment position are taken as indicator for the standard codon usage while CUG codons encoding serine and a preference for the polar and small amino acids serine, threonine, cysteine, and alanine are taken as indicator for the yeast alternative codon usage. However, the predictive power of the CUG positions is not equal. Positions important for protein folding, which are usually in the core of the proteins, and those important for protein interactions and ligand binding have a higher significance as those in loop regions at the protein surface. Therefore, we provide separate evaluations for the CUG codon position conservation and the amino acid similarity at CUG codon positions in the alignment. By analysing the genomes of yeast species with known CUG translation schemes, *Yarrowia lipolytica, Candida glabrata* and *Saccharomyces cerevisiae* using the Standard Code, and *Candida albicans, Debaryomyces hansenii* and *Lodderomyces elongisporus* using the AYCU, we found out that a simple majority rule is sufficient to predict the CUG codon translation scheme. Therefore, whatever the majority of the reference data is at a given CUG codon position, this will count for either the Standard or the AYCU. As a third option, the result is termed ambiguous or indiscriminative if the majority of the residues at CUG codon positions do not belong to either large hydrophobic residues (= > Standard Codon usage) or small polar residues (= > AYCU). The proposed CUG codon translation for the query sequence (genome or transcriptome assembly) will be given for every single reference protein and in summary for all reference data.

### Prediction of the tRNA_CAG_

Independent support for the proposed translation scheme is provided by tRNA prediction, which is performed with tRNAscan-SE. Subsequently, a BLAST search against reference leu-tRNA_CAG_ and ser-tRNA_CAG_ genes indicates the most probable identity of the tRNA_CAG_ in the query data. In addition, the predicted tRNA_CAG_ is aligned with the reference data for visual inspection.

### Translation check

Any given protein sequence can be checked for correct translation of CUG codons. This is an important option for all users who obtained protein sequences from databases, which did not resolve the codon translation yet. For example, at species genome project homepages gene annotations are often provided with non-uniform translations. All CUG codons are highlighted, differences between the correct and the given translation are indicated. If the given translation is partially incorrect, the correctly translated protein sequence can be downloaded in fasta-format.

### Case study

As example for the usability of Bagheera we choose *Candida bracarensis*, *Candida castellii*, *Candida maltosa*, *Candida nivariensis*, *Nakaseomyces bacillisporus*, and *Nakaseomyces delphensis* because these are not yet included in CyMoBase's reference data (Table 1). The genome assemblies were obtained from NCBI and uploaded into Bagheera. At NCBI, *C.bracarensis* and *C.nivariensis* are still grouped to the *Candida* branch (*mitosporic Saccharomycetales*) although a recent whole genome analysis showed their close homology to *Candida glabrata*, which belongs to the *Saccharomycetaceae*[40]. Therefore, we selected the "Candida albicans" feature set for *C.maltosa* and the "Saccharomyces cerevisiae" feature set for the other species for the gene prediction with AUGUSTUS. In all six genomes, homologs of almost all of the 38 reference proteins from *Candida albicans* and *Saccharomyces cerevisiae* were predicted (Table 1) supporting the completeness of the genome assemblies and similarity between the species. For *C.maltosa* 71 CUG codons were identified in 20 proteins, which is in agreement with the average number of CUG codons in the CTG clade (Mühlhausen and Kollmar, unpublished data). Accordingly, the genes of *C.bracarensis*, *C.castellii*, *C.nivariensis*, *N.bacillisporus*, and *N.delphensis* contain slightly more CUG codons (122 to 194 codons). In all five *Saccharomycetaceae*, about 75% of the CUG positions are conserved within the reference data. In *C.maltosa* about 50% of the CUG positions are conserved. Together, these data propose the AYCU for *C.maltosa* and the standard translation table for *C.bracarensis*, *C.castellii*, *C.nivariensis*, *N.bacillisporus*, and *N.delphensis*. AYCU for *C.maltosa* has already been shown [41].

**Table 1:** **Details of the CUG codon prediction in the genomes of six yeast species**

Species	Predicted proteins	Proteins with CUG	CUG codons	CUG position conservation		Sequence similarity		tRNA_CAG_	Accession number
				Std codon usage	AYCU	Std codon usage	AYCU		
*Candida bracarensis CBS 10154*	37	31	174	140	2	145	8	n.d.	CAPU00000000
*Candida castellii CBS 4332*	35	30	185	142	2	138	11	n.d.	CAPW00000000
*Candida maltosa Xu316*	34	20	71	4	35	18	14	ser-tRNA_CAG_	AOGT00000000
*Candida nivariensis CBS 9983*	34	30	182	131	2	150	7	n.d.	CAPV00000000
*Nakaseomyces bacillisporus CBS 7720*	33	25	122	89	3	100	2	n.d	CAPX00000000
*Nakaseomyces delphensis CBS 2170*	34	31	194	148	4	159	3	n.d.	CAPT00000000

### Limits of Bagheera

Possible limits of the tool might be that the query genes do not contain CUG codons and that the database contains only 18 protein families with 38 independent proteins. However, the proteins of most families of the reference data, e.g. myosins and dyneins, are very long and every of these proteins contains at least a few CUG codons (Figure 3). A whole genome sequence analyse will therefore always provide enough data for unambiguous assignment of the codon usage. Actins and tubulins also belong to the most widely used proteins for species phylogenies (e.g. recent analyses: [42–45]) and because of their high abundance in the cell it is highly likely that they are included in transcriptome assembly data and small-scale analyses. Although the presence of a leu-tRNA_CAG_ or ser-tRNA_CAG_ gene is a very strong indication for the Standard or AYCU, these genes are often not present in the genomes (e.g. in *Saccharomyces cerevisiae*) or might contain extremely long introns of more than 250 nucleotides hindering their identification and prediction.

## Conclusions

With this software we demonstrated that the most probable codon translation scheme for a given yeast genome can be determined by predicting motor and cytoskeletal proteins and comparing them to reference data. In total, 2071 sequences from 38 proteins belonging to 79 yeast species were included in the reference data providing a two-fold basis for the prediction of the most probable translation scheme: the amino acid composition at CUG positions and the conservation of CUG positions. The presence of hydrophobic amino acids in the reference data suggests the translation of the predicted CUG codons as leucine, while polar and small amino acids suggest their translation as serine. In addition, matching of CUG codons in the predicted genes with CUG codons in the reference data provides further support for the standard or alternative yeast codon usage. This information was implemented into a CUG codon prediction pipeline accessible via a web server called Bagheera. The predictive power of this implementation was demonstrated by a case study of the genomes of six *Saccharomycetes* species. In addition, the webserver offers the possibility to verify the translation of the CUG codons in any given protein sequence. Moreover, the webserver can be used as reference for the translation scheme used by individual yeast species.

## Availability and requirements

**Project name:** Bagheera – Predicting CUG codon translation in yeasts

**Project home page:**http://www.motorprotein.de/bagheera

**Operating system:** Platform independent

**Programming language:** Ruby

**Other requirements:** The current version of Bagheera has extensively been tested with Firefox version 15 or higher with JavaScript enabled, but should run on all modern browsers.

**Licence:** The source code for the web application and a command line tool can be obtained upon request and used under a Creative Commons License.

**Any restrictions to use by non-academics:** No.

## Electronic supplementary material

Additional file 1: **List of reference species.** This file contains a table with detailed information about the species, including teleomorph, anamorph and alternative scientific names, the species abbreviations as used throughout the web server, credits for the sequencing centres, and references to published genome analyses. In addition, the number of genes obtained from CyMoBase is listed. (XLS 62 KB)

Additional file 2: **CUG codon positions and amino acid composition of the reference data.** This table lists the CUG codon positions and the amino acid composition at respective alignment positions for every reference protein. The data are separated into two different sheets. In the first sheet, the positions of CUG codons are listed for every reference gene. The amino acid composition at every CUG position is included in the second sheet. (XLS 1 MB)

Additional file 3: **Number of CUG positions in the reference data and their conservation.** The table lists the number of CUG positions within each set of reference proteins in total counts and normalized to the protein lengths. In addition, the numbers of conserved CUG positions in at least two and five genes are given. The data are separated by reference species encoding CUG as leucine (sheet 1) and serine (sheet 2). These values are plotted in Figure 3. (XLS 30 KB)
